# Case Report: Tumor-to-tumor metastasis: a rare case of prostate adenocarcinoma metastasis to lung squamous cell carcinoma in a patient with multiple primary malignancies

**DOI:** 10.3389/fonc.2025.1605846

**Published:** 2025-06-09

**Authors:** Baoxiang Pei, Jikuan Liu, Zhiliang Hu, Fen Pan

**Affiliations:** Department of Thoracic Surgery, Jining No.1 People’s Hospital, Jining, China

**Keywords:** tumor-to-tumor metastasis, lung squamous cell carcinoma, lung lobectomy, prostate adenocarcinoma, prostatectomy

## Abstract

**Introduction:**

Tumor-to-tumor metastasis (TTM) is a rare occurrence in patients with two separate primary tumors, with the more malignant tumor more commonly metastasizing to a separate primary benign or low-grade tumor. Lung carcinomas are the most common metastatic tumor donors. However, the opposite phenomenon (lung carcinoma as a recipient of metastasis from prostate adenocarcinoma) is rarely previously reported in the literature. We report the case of TTM from prostate cancer to coexisting primary lung cancer.

**Case report:**

The 69-year-old male patient underwent surgery for tongue cancer on March 16, 2024, during which a lung mass was discovered in the right lower lung. The lung mass increased in size by follow-up thoracic computed tomography scan on October 4, 2024. Subsequently, the patient underwent single-port thoracoscopic right lower lung lobectomy and mediastinal lymph node dissection. Postoperative pathological results revealed that the lung mass had two components: lung squamous cell carcinoma and prostate cell carcinoma. MRI evaluation and PSA tests confirmed a prostate mass on November 8, 2024. Further, ultrasound-guided transperineal prostate biopsy was performed, and the biopsy pathology indicated prostate acinar adenocarcinoma. Subsequently, the patient underwent robot-assisted laparoscopic prostatectomy.

**Conclusions:**

This case report presents a rare and intriguing instance of TTM, specifically describing prostate adenocarcinoma metastasizing to a primary lung squamous cell carcinoma. The manuscript contributes valuable documentation to the limited literature on this rare phenomenon and is of potential interest to the oncology community.

## Background

Tumor-to-tumor metastasis (TTM) is defined as a metastasis between two true primary tumors, which is an extremely rare event in the medical field, with approximately 150 reported cases (1, 2). The phenomenon of TTM, which has been described for more than 100 years (3). According to the previous reports, donor tumors are usually malignant because of their high aggressiveness, while recipient tumors can be benign or malignant. The most common benign recipient tumor is meningioma, and the most prevalent malignant recipient tumor is renal cell carcinoma. Lung and breast carcinomas are most consistently the metastatic tumor donors (4–6). However, the opposite phenomenon (lung carcinoma as a recipient of metastasis from prostate adenocarcinoma) is rarely previously reported in the literature. We conducted an extensive literature search. Hamadi R (7), Liu S (8) and Pei R (9) et al. had separately reported the rare case of TTM, which was prostate cancer metastasizing to primary lung adenocarcinoma. Here, we report the first case of prostate adenocarcinoma metastasizing to primary lung squamous cell carcinoma. The difference from the previously reported literature is the pathological type of the recipient tumor.

## Case report

The Chinese patient, a 69-year-old male, had a 20-year history of hypertension and was diagnosed with pulmonary tuberculosis four years ago, which was clinically cured. On March 16, 2024, he underwent left tongue and oropharyngeal malignant tumor resection, cervical lymphadenectomy, left forearm vascularized free flap reconstruction, and arterial anastomosis. Pathological results indicated moderately differentiated squamous cell carcinoma at the left tongue base and oropharyngeal primary site, with a tumor size of approximately 3.5×2×2cm; no metastatic cancer was found in the surrounding lymph nodes (0/19) from the cervical lymphadenectomy.

**Figure 1:**
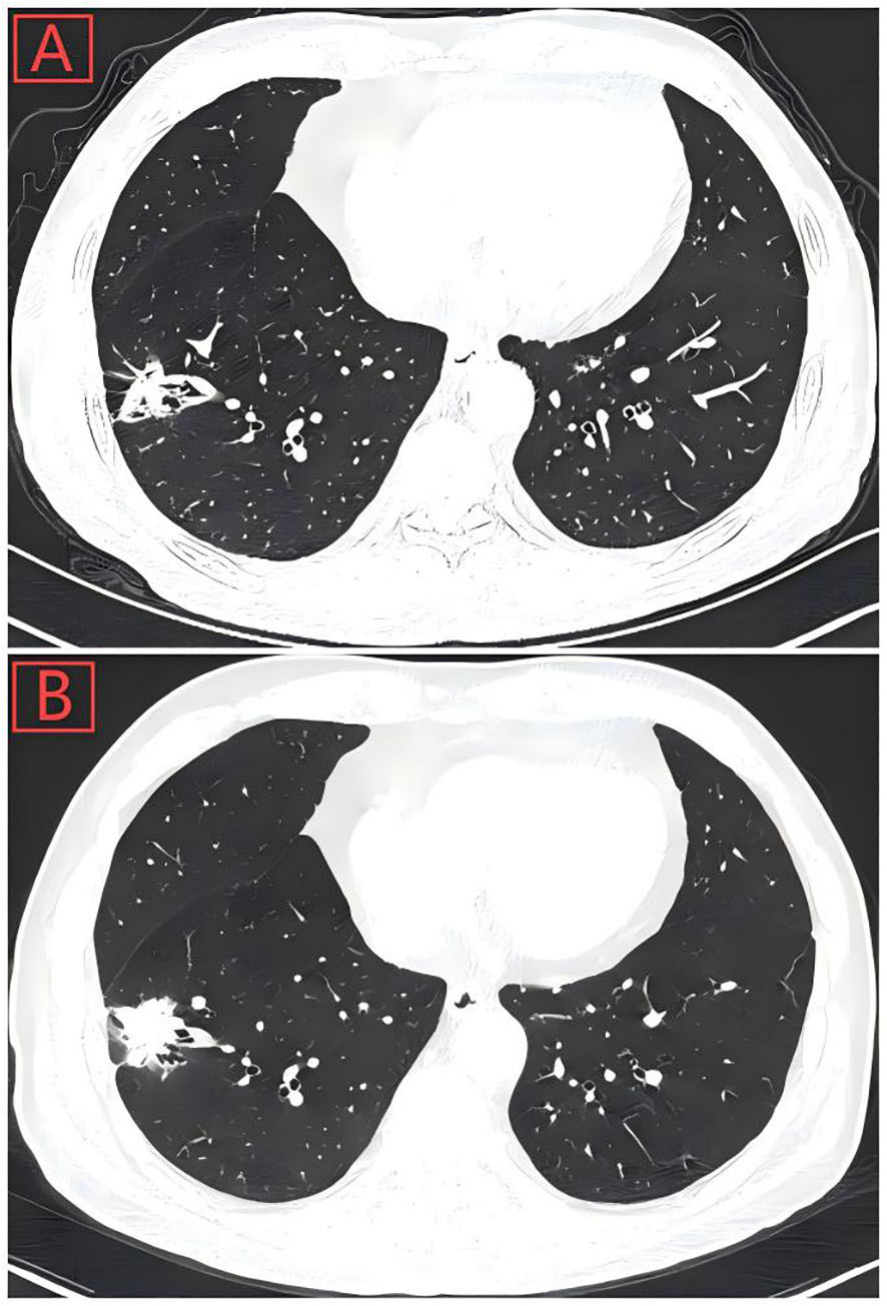
**(A)** Thoracic computed tomography scan showed a pulmonary nodule with solid opacity (28-mm diameter) on March 16, 2024, located in the peripheral aspect of the right lower lobe. **(B)** Pulmonary nodule had increased in size (35-mm diameter) by follow-up Chest CT on October 4, 2024.

**Figure 2:**
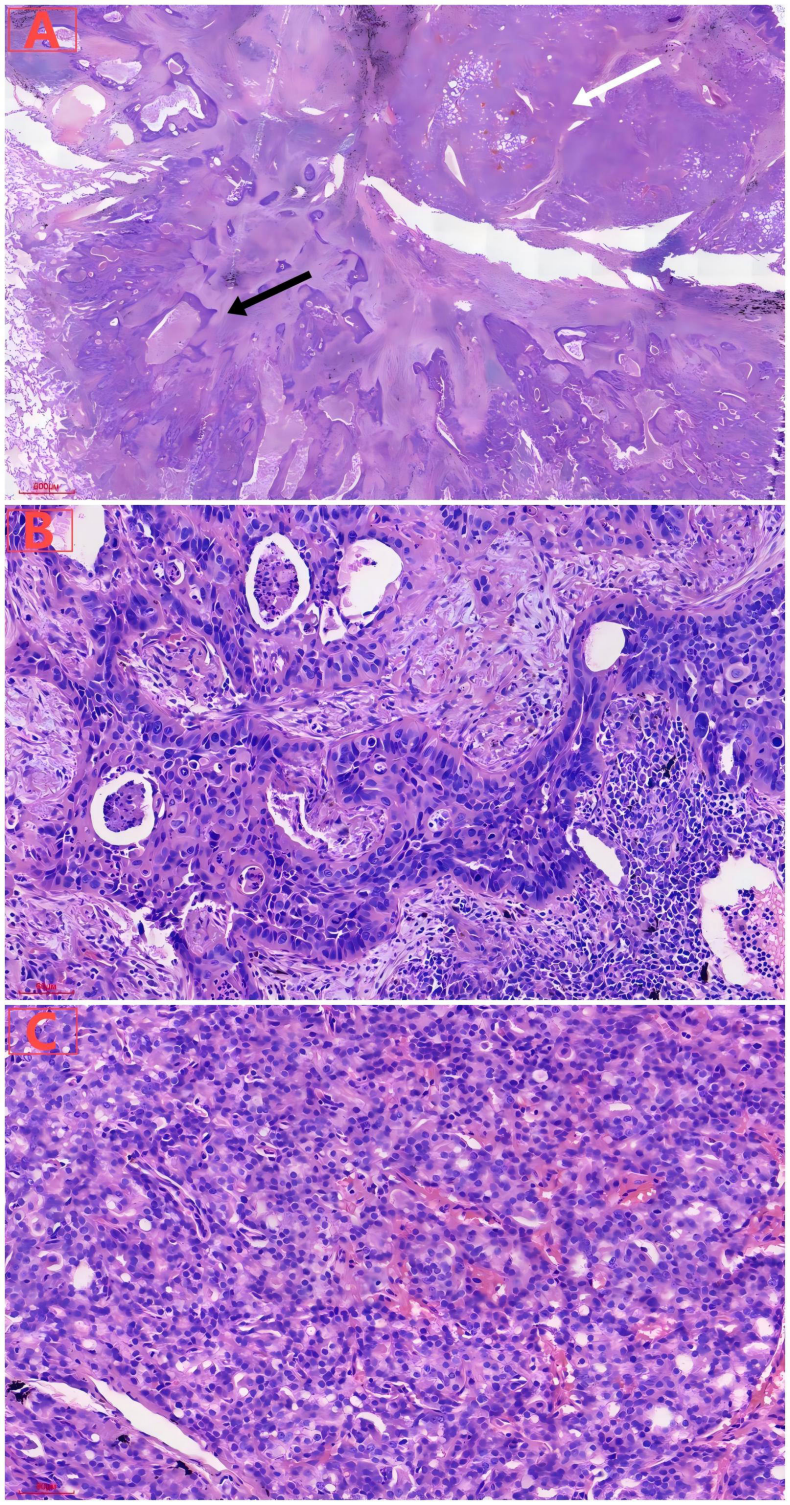
**(A)** Under low magnification, lung tumor consisted of cancer cells with two different histological structures and cytological appearances, including lung squamous carcinoma components (black arrow) and prostate adenocarcinoma components (white arrow). **(B)** Under high magnification, lung squamous cell carcinoma showed solid nests of cells, with visible necrosis. **(C)** Under high magnification, metastatic prostate acinar adenocarcinoma exhibited tubular arrangement with moderate cellular atypia.

**Figure 3:**
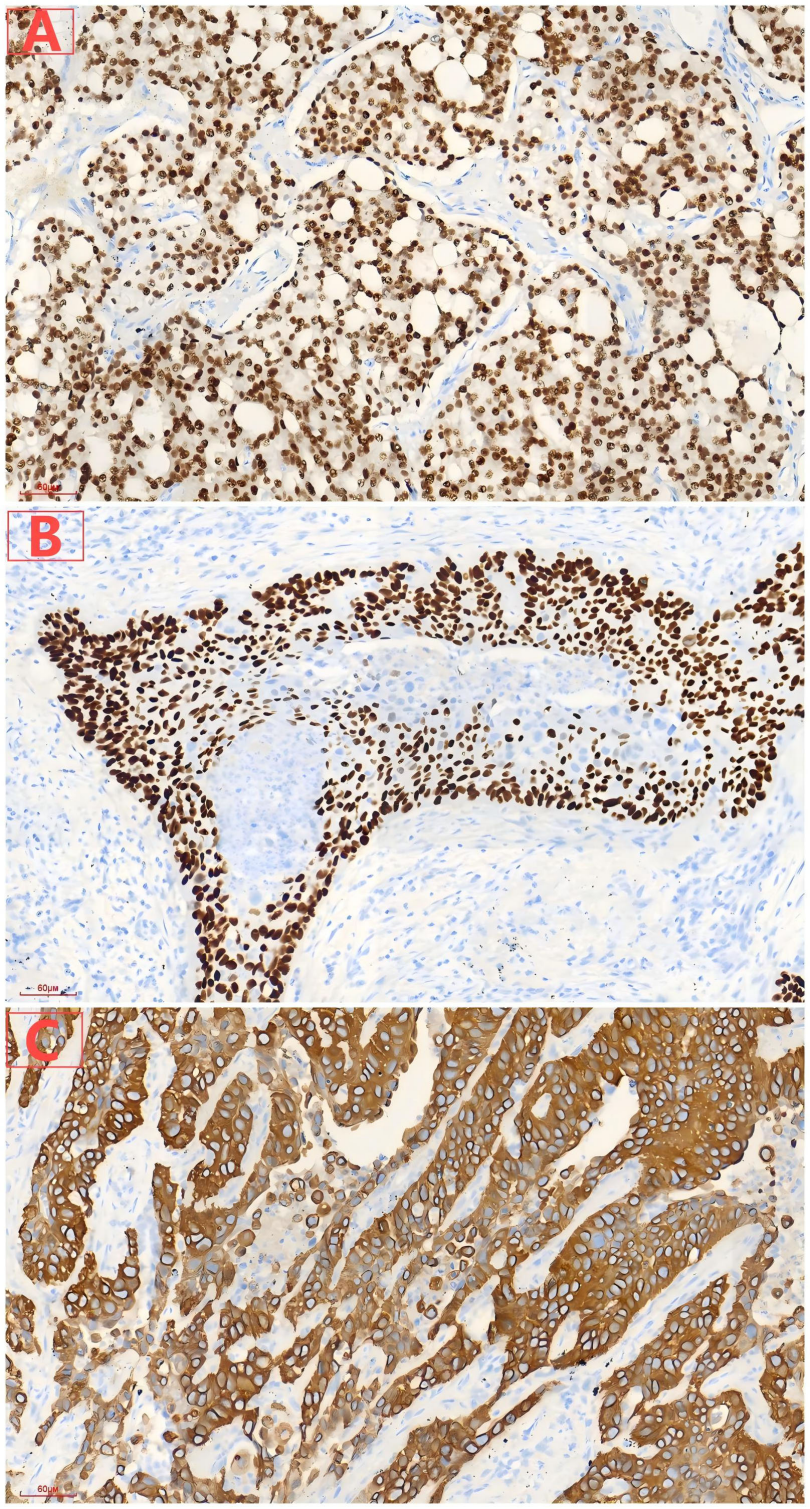
**(A)** Diffuse expression of NKX3.1 in prostatic acinar adenocarcinoma. **(B)** Diffuse expression of P63 in lung squamous cell carcinoma. **(C)** Diffuse expression of CK5/6 in lung squamous cell carcinoma.

During the tongue cancer surgery, a mass was found in the right lower lung, which increased in size by follow-up thoracic computed tomography scan on October 4, 2024 (**Figure 1**). On October 11, 2024, carcinoembryonic antigen, cytokeratin 19 fragments, squamous cell carcinoma-related antigen, and gastrin-releasing peptide precursor were all normal. On October 14, 2024, an electronic bronchoscopy revealed a granulomatous neoplasm at the site of the main trachea’s original incision, which was treated with electrocautery, and the neoplasm was sent for pathology. The biopsy specimen from the neoplasm in the main trachea showed inflammatory granulation tissue covered by squamous epithelium. On October 18, 2024, the patient underwent single-port thoracoscopic right lower lung lobectomy and mediastinal lymph node dissection. Postoperative pathological results indicated moderately differentiated squamous cell carcinoma in the right lower lung nodule, with a tumor size of approximately 3.5×2×1.8 cm, without invasion of the pleura, and glandular structures with cellular atypia were observed, consistent with prostate adenocarcinoma metastasis, with the maximum diameter of the metastatic focus measuring approximately 0.3 cm (**Figure 2**). The sent lymph nodes from groups 2, 3, 4 (0/4), group 7 (0/3), and group 11 (0/2) showed no metastatic cancer. Immunohistochemical (IHC) results: Lung squamous cell carcinoma: CK5/6(+), P40(+), P63(+), CK7(-), Napsin A(-), TTF-1(-), CD56(-), CgA(-), Syn(-), SSTR2(focal+), SMARCA4(+), INI-1(+), CK(+), CK8/18 (partial+), Vimentin(-), EMA(+), PAX-8(-), PSA(-), NKX3.1(-), Ki67(+, about 15%). Prostate cancer: NKX3.1(+), PSA(+), CK5/6(-), P40(-), P63(-), CK7(-), Napsin A(-), TTF-1(-), CD56(-), CgA(-), Syn(weak+), INSM1(weak+), SSTR2(-), SMARCA4(+), INI-1(+), CK(+), CK8/18(+), Vimentin(-), EMA(-), PAX-8(-), Ki67(+, about 10%). IHC results showed that the lung cancer cells were positive for CK5/6 and P63, but negative for PSA and NKX3.1. And the prostate cancer cells were positive for NKX3.1, whereas negative for CK5/6 and P63 (**Figure 3**).

**Figure 4:**
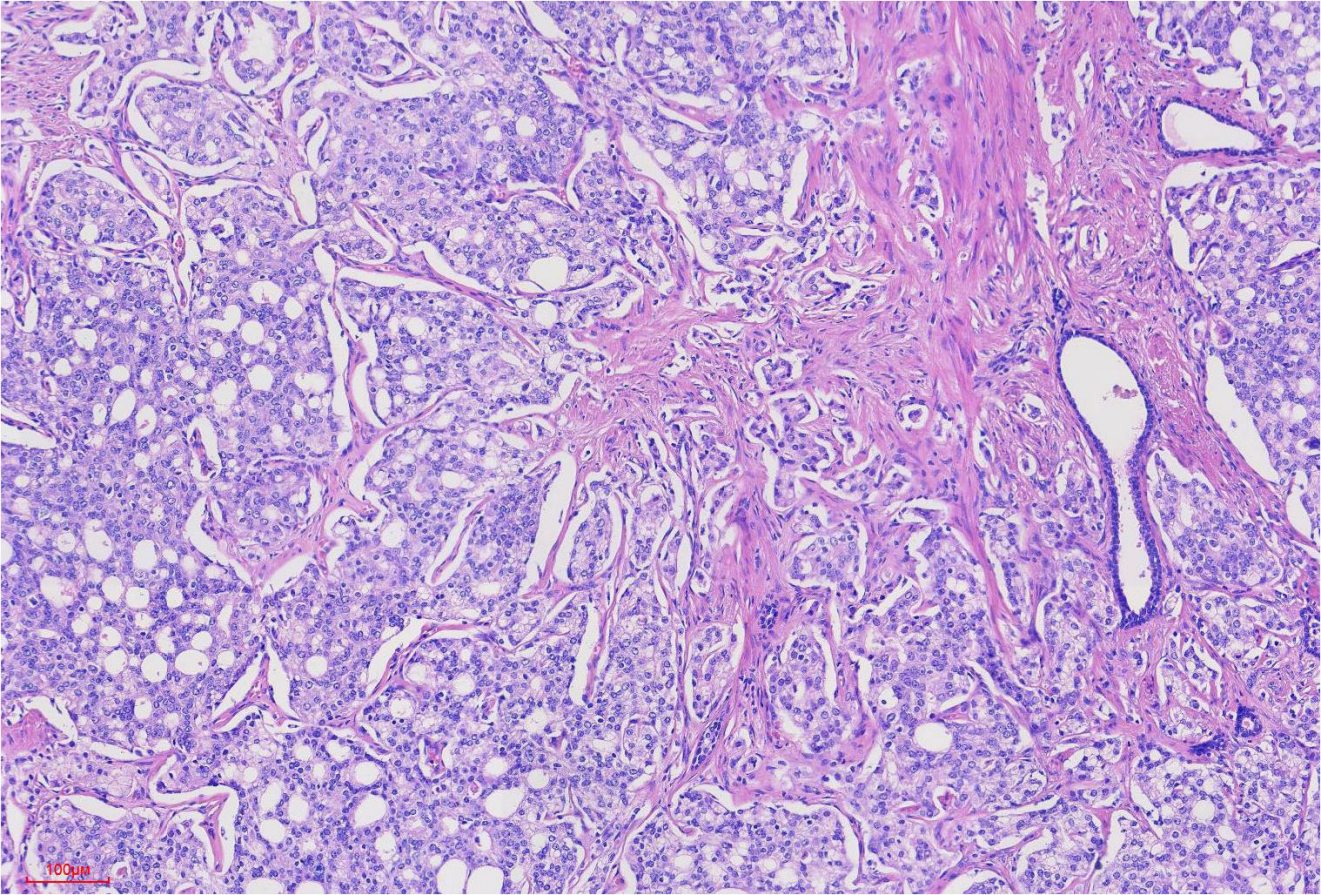
Prostate cancer tumor cells were arranged in tubular and sieve-like patterns, with Gleason score: 4 + 4 = 8 points.

During the lung cancer surgery, prostate adenocarcinoma metastasis was found within the lung squamous cell carcinoma lesion. On November 8, 2024, prostate magnetic resonance imaging (MRI) showed abnormal signals in the prostate, suggesting prostate cancer and prostatic hyperplasia. On November 8, 2024, total prostate-specific antigen (T-PSA) was 90.42 ng/ml, free prostate-specific antigen (F-PSA) was 3.33 ng/ml, and the F-PSA/T-PSA ratio was 0.04. On November 9, 2024, ultrasound-guided transperineal prostate biopsy was performed, and the biopsy pathology indicated prostate acinar adenocarcinoma. On November 13, 2024, the patient underwent robot-assisted laparoscopic prostatectomy under general anesthesia, and postoperative pathology indicated prostate acinar adenocarcinoma in the prostate-seminal vesicle tissue (**Figure 4**), with a Gleason score of 4 + 4 = 8 (International Society of Urological Pathology Grade Group 4), with tumor occupying about 70% of the prostate tissue, tumor invading nerves; the apex margin and focal periphery showed cancer involvement, while the base margin showed no cancer involvement; bilateral vas deferens margins and bilateral seminal vesicles showed no cancer involvement; lymph nodes in the surrounding fat (1/1) showed metastatic cancer. No disease progression or recurrence has been observed based on the current situation. The patient has been followed up for 6 months postoperatively and remains disease-free. The patient has given informed consent for the publication of this case report.

## Discussion

Heterogeneous multiple primary malignancies (MPM) refer to the occurrence of two or more different types of primary malignant tumors in a patient at the same time or at different times. These tumors may originate from the same or different tissues, and their pathogenesis and biological characteristics may differ significantly (10). With a deeper understanding of tumor biology, it has been found that some patients can have two or more different types of tumors in the same anatomical location, meaning that one tumor lesion contains metastatic components of other types of tumors; this phenomenon is referred to as TTM, and its mechanism has not been fully elucidated (1, 2). Richter et al. (11) proposed two main criteria to distinguish tumor-to-tumor metastasis from collision tumors, which include the presence of tumor foci with different histology within the host tumor and the presence of metastatic primary cancer. The patient’s postoperative pathology of tongue cancer showed moderately differentiated squamous cell carcinoma with no metastasis in the surrounding lymph nodes, not advanced tongue cancer with lung metastasis or advanced lung cancer. Postoperative pathological results revealed two distinct components within the lung mass: moderately differentiated squamous cell carcinoma and prostate adenocarcinoma. The presence of both histological structures was confirmed by immunohistochemical (IHC) staining. The lung cancer cells were positive for CK5/6, P63, and negative for PSA and NKX3.1, while the prostate cancer cells were positive for NKX3.1 and PSA, and negative for CK5/6 and P63. These findings strongly support the diagnosis of TTM rather than adenosquamous carcinoma or a collision tumor. The absence of metastatic cancer in the surrounding lymph nodes further supports the diagnosis of TTM. The diagnosis of prostate adenocarcinoma was also supported by MRI, serologic examination and puncture biopsy. In contrast to the lung adenosquamous carcinoma, neither the histological structure nor the immunohistochemical features of the tumor in this case supported the diagnosis of adenosquamous carcinoma.

The phenomenon of TTM, which has been described for more than 100 years (3). The donor tumor can be any malignant tumor, while the recipient tumor can be benign or malignant, with benign meningiomas being more common and malignant renal cell carcinoma being more prevalent (4–6). According to literature reviews, TTM with prostate cancer as the donor is extremely rare, with only sporadic case reports documented to date. **Table 1** is a summary of previously reported cases and an analysis of key differences (3, 7–9, 12–15). We conducted an extensive literature search and found only three similar cases of prostate cancer metastasizing to lung adenocarcinoma (7–9). Our case represents the first documented instance of prostate adenocarcinoma metastasizing to lung squamous cell carcinoma. This unique case highlights the importance of considering TTM in the differential diagnosis of patients with multiple primary malignancies. The difference from the previously reported literature is the pathological type of the recipient tumor. This phenomenon not only affects the treatment strategy for the patient but also has significant implications for prognostic assessment. Although the current treatment outcomes appear promising, the treatment effects still require long-term therapeutic observation.

**Table 1:** Previously reported cases of prostate cancer as donor in tumor-to-tumor metastasis (TTM).

Reference	Recipient Tumor	Histological Pathology	Age	Treatment	Outcome
Hamadi R (7)	Lung adenocarcinoma	NKX3.1+, PSA+, PSMA+ prostate cancer coexisting with lung cancer mass	80y	Prostate: Prostatectomy + adjuvant radiotherapy + bicalutamide + leuprolide + darolutamide Lung cancer: Lobectomy	Unknown
Liu S (8)	Lung adenocarcinoma	NKX3.1+, PSA+ prostate cancer coexisting with lung cancer mass	58y	EGFR TKI amitinib and endocrine therapy with bicalutamide and leuprorelin	No disease progression at 7 months
Pei R (9)	Lung mucinous adenocarcinoma	NKX3.1+, PSA+ prostate cancer coexisting with lung cancer mass	71y	Prostate: Prostatectomy Lung cancer: Chemotherapy and radiation therapy	No disease progression at 6 months
Cavalcante A (12)	Chromophobe renal cell carcinoma (RCC)	Prostate adenocarcinoma (PSA+) within RCC	80y	Prostate: ADT RCC: Nephrectomy	Unknown
Moody P (3)	Meningioma	PSA+ prostate cancer cells in meningioma	58y	Prostate: Radiotherapy + Chemotherapy Meningioma: Craniotomy for tumor resection	Unknown
Neville IS (13)	Transitional meningioma	PSA+ prostate cancer cells in meningioma	68y	Craniotomy for tumor resection	Death at 2 months (septic shock from pneumonia)
Pugsley D (14)	Meningothelial meningioma	PSA+ prostate cancer cells in meningioma	70y	Prostate: ADT + goserelin Meningioma: dexamethasone + Craniotomy for tumor resection + Radiotherapy	Survival > 3 years
Furia S (15)	Type A thymoma	PSA+ prostate cancer cells in thymoma	75y	Prostate: ADT + Chemotherapy Thymoma: thymectomy	Unknown

PSA, Prostate-Specific Antigen; ADT, Androgen Deprivation Therapy; PSMA, Prostate-Specific Membrane Antigen.

Metastasis of a malignant tumor to another primary tumor is a rare phenomenon. There are few possible explanations for this phenomenon, including some anatomical considerations (high blood flow) (16, 17), a favorable microenvironment in the primary tumor that can accommodate metastatic tumor cells (7, 18, 19), and patient characteristics, including personal history of multiple synchronous or metachronous tumors (19). In this case, the presence of metastatic prostate adenocarcinoma within lung squamous cell carcinoma suggests a unique interaction between the two tumor types. The high vascularity and specific growth factors in the lung squamous cell carcinoma microenvironment may have facilitated the engraftment of prostate cancer cells. This contrasts with the more common scenario where lung adenocarcinoma serves as a recipient, highlighting the importance of tumor-specific microenvironments in TTM.

Clinically, the diagnosis of TTM often relies on imaging examinations and tissue biopsies. In patients with lung squamous cell carcinoma, if prostate adenocarcinoma metastatic components are found, treatment plans should be comprehensively considered, including targeted therapy and immunotherapy. Moreover, recognizing the phenomenon of TTM helps to more comprehensively assess the patient’s tumor burden. By involving experts from different disciplines, the patient’s condition can be evaluated from multiple perspectives, leading to more comprehensive and personalized treatment plans. Additionally, with the development of new technologies such as liquid biopsy and genomic analysis, more accurate tumor characteristic information can be provided to assist clinicians in formulating personalized treatment strategies (20). Recent studies have highlighted the role of specific signaling pathways in tumor metastasis. For instance, Ononin has been shown to inhibit tumor bone metastasis and osteoclastogenesis by targeting the mitogen-activated protein kinase (MAPK) pathway in breast cancer (21). Similarly, gradient rotating magnetic fields have been reported to impair F-actin-related gene CCDC150, thereby inhibiting triple-negative breast cancer metastasis by inactivating the TGF-β1/SMAD3 signaling pathway (22). These advancements highlight the potential for novel therapeutic approaches in managing complex metastatic diseases. However, the current literature in this field is still insufficient, and more high-quality studies are needed to fill this gap.

This case report is limited by its single-case nature, highlighting the need for larger, registry-based studies to better understand the true incidence of TTM. Additionally, emerging molecular and genomic tools, such as liquid biopsy and single-cell sequencing, may offer new insights into the detection and management of occult TTM in other clinical contexts. These technologies could potentially identify early metastatic events and guide more personalized treatment strategies in the future.

In conclusion, We present the first documented case of prostate adenocarcinoma metastasizing to primary lung squamous cell carcinoma. This rare phenomenon of tumor-to-tumor metastasis (TTM) underscores the importance of comprehensive pathological evaluation and multidisciplinary collaboration in managing patients with multiple primary malignancies. Recognizing TTM can significantly impact diagnostic strategies, staging, and treatment decisions. By combining a multidisciplinary comprehensive treatment model, we aim to enhance understanding of such diseases and provide references for individualized treatment plans. Future research should further explore the mechanisms underlying TTM to improve patient prognosis and optimize treatment strategies.

## Data Availability

The raw data supporting the conclusions of this article will be made available by the authors, without undue reservation.
